# Progress in Phototherapy and Electro-Therapeutics

**Published:** 1903-10-17

**Authors:** 


### Progress in Phototherapy and Electro-Therapeutics.

(*Concluded from page* 34.)

**Electricity and High-frequency Currents.**—Macintyre [38] found that the high-frequency currents were instrumental in completely relieving severe neuralgia of the second division of the fifth nerve. After months of intense suffering, the teeth having been removed as well as the alveolar process without relief, all pain disappeared after three weeks' treatment with Oudin's electrode, locally applied. No recurrence occurred in six months. In a second case, however, though the pain was relieved for a year, the recurrence of it could not be relieved.

A new feature in the use of currents of high-frequency is introduced by Cunningham Bowie.[39] By specially arranged apparatus he can vary the frequency or rate of oscillation within the limits of 50 to 80,000 per second; and at the same time can provide currents of high frequency and low potential (from 40 to 100 volts). The inventor claims that his method gives a higher magnitude of current; thus the weak current runs up to 100 milliamps.; medium to 1,000 milliamps.; strong currents to 2–3 amps. The apparatus consists of a step-up transformer, an oil-immersed condenser, and a spark gap. The condenser armatures are connected by a flat helix which induces currents in a second helix arranged on an adjustable slide. The patient is placed in a circuit connected with this secondary helix and obtains greater or less voltage according as whether the secondary helix is close or far from the primary. The frequency rate can be varied, by altering the capacity of the condenser, arranged for by sliding the condenser plates to or from one another.

The author states that by lessening the voltage, large ampèrage at high frequency can be administered without pain, and, in the case of tuberculous pulmonary conditions, without hæmorrhage which cannot be done with currents of high voltage. No risk is incurred in this way while using the highest possible ampèrage and frequency though the sitting last 10–15 minutes. Together with intra-laryngeal injections of antiseptics, he has seen the most satisfactory results.

Another apparatus which is described is known as Nodon's valve,[40] which is a rectifier for alternating currents. It is used when it is desired to store batteries, drive motors, or excite an *x* ray coil from an alternating circuit. It is noiseless, without smell, and requires no attention, and yet is effective. It is based upon the fact that aluminium will allow a current to pass when it is the negative pole of a cell, whilst when it is the positive pole it is practically a non-conductor. "By an ingenious combination of four cells (of iron and aluminium poles) properly connected, it is possible to utilise both the semi-phases of the alternating current and thus to approximate very closely to a continuous current." The fluid used in the cells is ammonium phosphate, and the cells are of sheet iron externally and hollow tubes of aluminium alloyed with zinc internally. A current of air is driven by a small motor through the hollow tubes. In this way there is practically no heat, and surface evaporation is the only loss. There is apparently very little corrosion of the iron and aluminium poles.

Fox [41] treats rodent ulcer by means of the electricity derived from Stohrer's bichromate batteries. Needles are inserted into the surrounding healthy tissue under general anæsthesia, and a current of 300–1,000 milliamps. at a voltage of 40–50 is delivered through the ulcer for a second or two and then reversed. This is done with the needles about half an inch apart till the whole has been encircled, and then in three to six days a slough separates, leaving healthy granulations. After electricity there is no pain and no hæmorrhage, and the cicatrix he claims to be less than after excision, and there is less risk of recurrence.

Sloan[42] has been watching the effect of alternating currents on 67 patients of different kinds. These cases comprise atonic dyspepsia, visceral neuroses, neuro-muscular asthenia, asthma, lumbo-pelvic neuritis, persistent vomiting with or without diarrhœa, peripheral vaso-motor disturbances (paretic or spasmodic), neurasthenia, uterine atony and angina pectoris. He believes that in the great majority of cases, within certain limits of course, the kind of electric energy employed is of little consequence if only the dose be carefully measured and regulated to suit the nature of the case. He holds that electricity acts as a stimulant and tonic to nerve tissue, improving its nutrition. An instrument to measure the current is a *sine quâ non* if the treatment is to be carried out on scientific lines. He believes the sympathetic ganglia to be directly stimulated. His method of application is by means of large moist clay electrodes, the positive one 9 × 6 between the shoulder blades, and the negative 9 × 10 ins. over the epigastrium. The current is from a secondary coil of 8,000 turns of fine wire, and the dose measured by his own Faradimeter. With the sinusoidal current the alternations amount to 1,800 per minute. The dose at the first treatment is 2 to 3 milliamps, gradually increased to 7 to 8 milliamps. for 15 minutes' duration. Rest is enjoined after treatment. The sittings are given every second, third or fourth day, according to the case, and six to eight are required.

Commenting upon Aspinall's paper on electric shocks, of which a *resumé* has been given in this journal, Dr. S. Jellinck[43] states that, as far as susceptibility is concerned, women and children offer less resistance than men; and mucous surfaces are more dangerous than skin. Diseases which lower the skin resistance, *e.g.*, Basedow's disease, cardiac and renal, and some skin diseases, predispose to accident. With regard to chloroform and sleep, high tension interrupted currents are not only harmless for rabbits during deep narcosis, but are even life-saving.

The left side is more dangerous than the right, owing to the ganglia of the heart.

Burns lower the resistance of the body, and the longer tissue is exposed to the current the lower the resistance becomes. This is important when it is considered that the formula A × V × T is not constant when a man is concerned, for as resistance decreases ampérage increases: the longer the time the more the current. Alternating have not entirely the same value as direct currents, though 500–600 volts are dangerous, and 1,000 fatal in both, yet with low tension alternating current that of 100–200 volts is much more disagreeable than direct. The direct current increases 10–20 millimetres, and the alternating decreases the blood pressure 10–15 millimetres. To restore those who have been shocked they should not be held up "to flush the brain," as in the central nervous system he has found hæmorrhage in the grey matter and alterations in the ganglion cells, and this proceeding would rather aid such conditions. Venesection might be tried; and artificial respiration, massage of the abdomen and precordia, hot bath, and ether, camphor, and strychnine subcutaneously.

[38] Brit. Med. Jour., June 6. [39] Lancet, May 2. [40] Ibid., April 18. [41] Brit. Med. Jour., Jan. 3. [42] Lancet, May 30. [43] Ibid., Feb. 7.

